# Percutaneous endoscopic lumbar discectomy *via* the medial foraminal and interlaminar approaches: A comparative study with 2-year follow-up

**DOI:** 10.3389/fsurg.2022.990751

**Published:** 2022-11-02

**Authors:** Sen Huang, Zhenfei Wang, Long Xu, Jinhui Bu, Bo He, Mengjiao Xia, Tao Chen, Juan Gao, Guangpu Liu, Ru Niu, Chao Ma, Guangwang Liu

**Affiliations:** ^1^ Department of Emergency Surgery, Jiangsu Province Hospital on Integration of Chinese and Western Medicine, Nanjing, China; ^2^ Department of Orthopedic Surgery, Xuzhou Central Hospital, Xuzhou Clinical School of Xuzhou Medical University, Xuzhou Central Hospital Affiliated to Nanjing University of Chinese Medicine, The Xuzhou School of Clinical Medicine of Nanjing Medical University, Xuzhou Central Hospital Affiliated to Medical School of Southeast University, Xuzhou, China; ^3^ Department of Orthopedic Surgery, Graduate School of Bengbu Medical College, Bengbu, China; ^4^ Department of Orthopedic Surgery, Xuzhou Clinical School of Xuzhou Medical University, Xuzhou, China

**Keywords:** lumbar disc herniation, minimally invasive, percutaneous endoscopic interlaminar discectomy, percutaneous endoscopic medial foraminal discectomy, ligamentum flavum

## Abstract

**Objective:**

The purpose of this study was to analyze the clinical effect of percutaneous endoscopic medial foraminal discectomy (PEMFD) in the treatment of lumbar disc herniation (LDH).

**Methods:**

We retrospectively examined and compared clinical data from 39 single-level LDH patients who underwent PEID and 47 who underwent PEMFD. All the patients were diagnosed with single-level LDH and were treated in Xuzhou Central Hospital for single-segmental lumbar disc herniation between June 2017 and December 2019. Collect and count surgical-related indicators, intraoperative bleeding volume and 24-hour postoperative drainage volume, lower extremity numbness Visual Analogue Scale (VAS), the pain VAS and lumbar Oswestry Disability Index (ODI) scores.

**Results:**

Intraoperative bleeding volume and 24-hour postoperative drainage volume were significantly lower in the PEMFD group (*p* < 0.05). Operation time and length of hospital stay did not significantly differ between the groups. Transient spinal cord injury and surgical site infection did not occur. Recurrence occurred in two patients in each group. Repeat surgery in these patients demonstrated remarkable epidural scarring in the PEID group patients; no scarring was evident in the PEMFD group patients. The numbness VAS score 72 h after surgery and the pain VAS and ODI scores 1 month after surgery significantly differed between groups; however, pain VAS and ODI scores 6, 12, and 24 months after surgery did not. At last follow-up, the modified MacNab criteria outcome did not significantly differ between the groups.

**Conclusion:**

PEMFD and PEID have similar short- and medium-term outcomes. However, PEMFD has several advantages: simplicity, lower bleeding volume, and preservation of the LF.

## Introduction

Lumbar disc herniation (LDH) is the most common cause of low back pain and sciatica (1). Its incidence has been increasing and affected patients are becoming younger. Although conventional open surgery for LDH generally has a good outcome, it is relatively traumatic to local structures and can be associated with significant postoperative complications and prolonged recovery (2). Endoscopic LDH surgery is a minimally invasive surgical option (3). Spinal endoscopy was introduced in the 1990s and has become a widely accepted technique for treating LDH (4). The transforaminal and interlaminar approaches are common approaches used in percutaneous endoscopic lumbar discectomy. Percutaneous endoscopic interlaminar discectomy (PEID) (5) has multiple advantages, including its minimally invasive nature, small incision, and rapid recovery. It can achieve the same clinical effect as conventional open surgery (6, 7). However, during PEID, a portion of the ligamentum flavum (LF) is exposed and then stripped to allow placement of a working cannula into the spinal canal. After placement, the dural sac and nerve root are explored endoscopically and the herniated nucleus pulposus is identified and removed. Bleeding may occur from the abundant venous plexus on the surface of the dural sac during exploration. The LF serves as a natural barrier of the spinal canal and plays a role in protecting the dural sac. Its preservation during surgery can prevent postoperative adhesions, decrease iatrogenic stimuli to the dural sac, and reduce the incidence of epidural fibrosis (8–12, 20).

The LF can be preserved during surgery using a variety of methods (13, 14). Percutaneous endoscopic medial foraminal discectomy (PEMFD), which is a modification of the conventional interlaminar approach, is one such technique that does not expose the nerve and dural sac. With this approach, the LF attached to the articular process can be separated and detached in the medial foraminal segment and then pushed medially along with the nerve and dural sac into the spinal canal to expose the herniated nucleus pulposus. PEMFD has a short learning curve, is not technically difficult, and can be performed relatively rapidly. Most importantly, it is associated with minimal manipulation of the nerve and dural sac, and consequently lower risk of iatrogenic injury to these structures. This study aimed to investigate and compare the clinical outcomes of PEID and PEMFD in patients with single-level LDH.

## Data and methods

### Patients

Eighty-six patients (49 men and 37 women; age range, 29–78 years) diagnosed with single-level LDH who failed at least six months of conservative treatment and underwent endoscopic surgical treatment in Xuzhou Central Hospital between June 2017 and December 2019 were retrospectively examined. We excluded patients with lumbar spine instability, multilevel pathology on imaging, a history of open lumbar spine surgery, LF hypertrophy or calcification, lumbar spinal stenosis (moderate and severe spinal stenosis caused by other reasons), or infection. Thirty-nine patients underwent PEID and 47 underwent PEMFD. Patient characteristics, including age, gender, and lesion site, did not significantly differ between groups (Table 1). All patients were followed for at least 24 months. The study was conducted in accordance with the Declaration of Helsinki. All patients provided written informed consent.

**Table 1:** Patient characteristics.

General data	PEID group	MF-PELD group	Statistics	*p*
Case (*n*)	39	47	–	–
Gender (*n*)				
Male	23	26	*χ*^2 ^= 0.116	0.733
Female	16	21		
Age (years, *x* ± *s*)	46.36 ± 13.64	46.70 ± 12.00	*t* = 0.124	0.902
Lesion segment (*n*)				
L3/4	10	8	*χ*^2 ^= 1.231	0.540
L4/5	16	19		
L5/S1	13	20		

### Surgical procedure

A single surgeon performed all procedures.All patients received local anesthesia with 0.5% lidocaine 4–8 ml.

**Figure 1:**
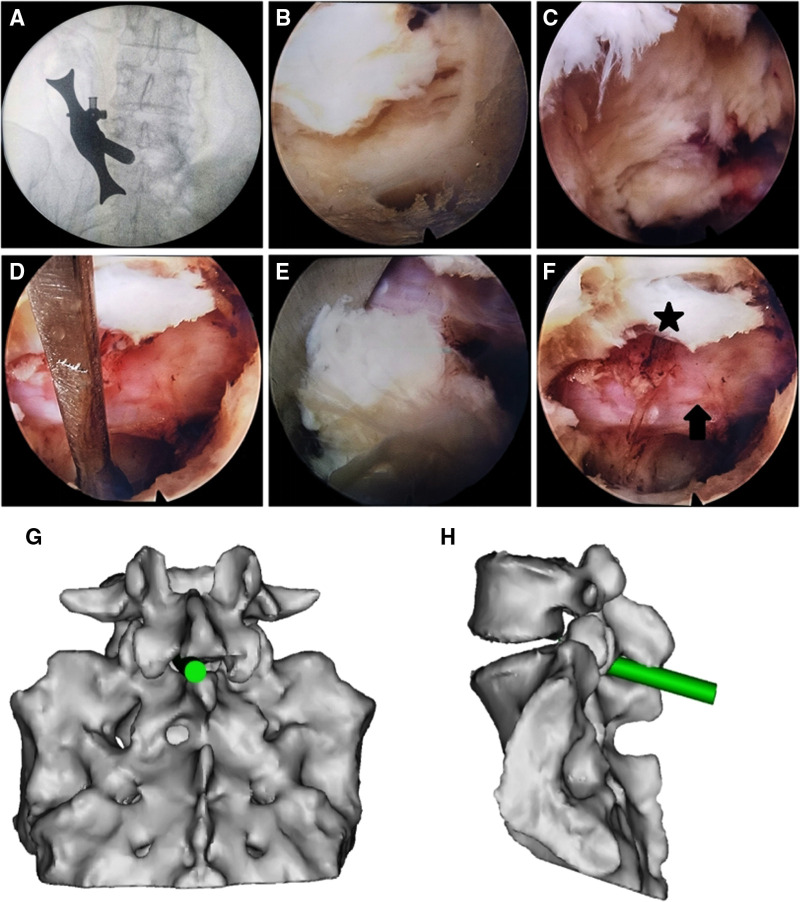
Percutaneous endoscopic lumbar discectomy *via* the interlaminar approach in a 37-year female with L5–S1 disc herniation. (**A**) The dilator was localized using fluoroscopy before surgery and placed as close to the ligamentum flavum as possible. (**B**) The ligamentum flavum after removing fat and muscle tissue from its surface. (**C**) Exposure of the dural sac and nerve root by opening the ligamentum flavum. (**D**) Release of the nerve root. (**E**) Herniated nucleus pulposus. (**F**) Intraoperative S1 nerve root (arrow), and the opened ligamentum flavum (star). (**G,H**) Schematic presentations of the working cannula in frontal (**G**) and side (**H**) views.

**Figure 2:**
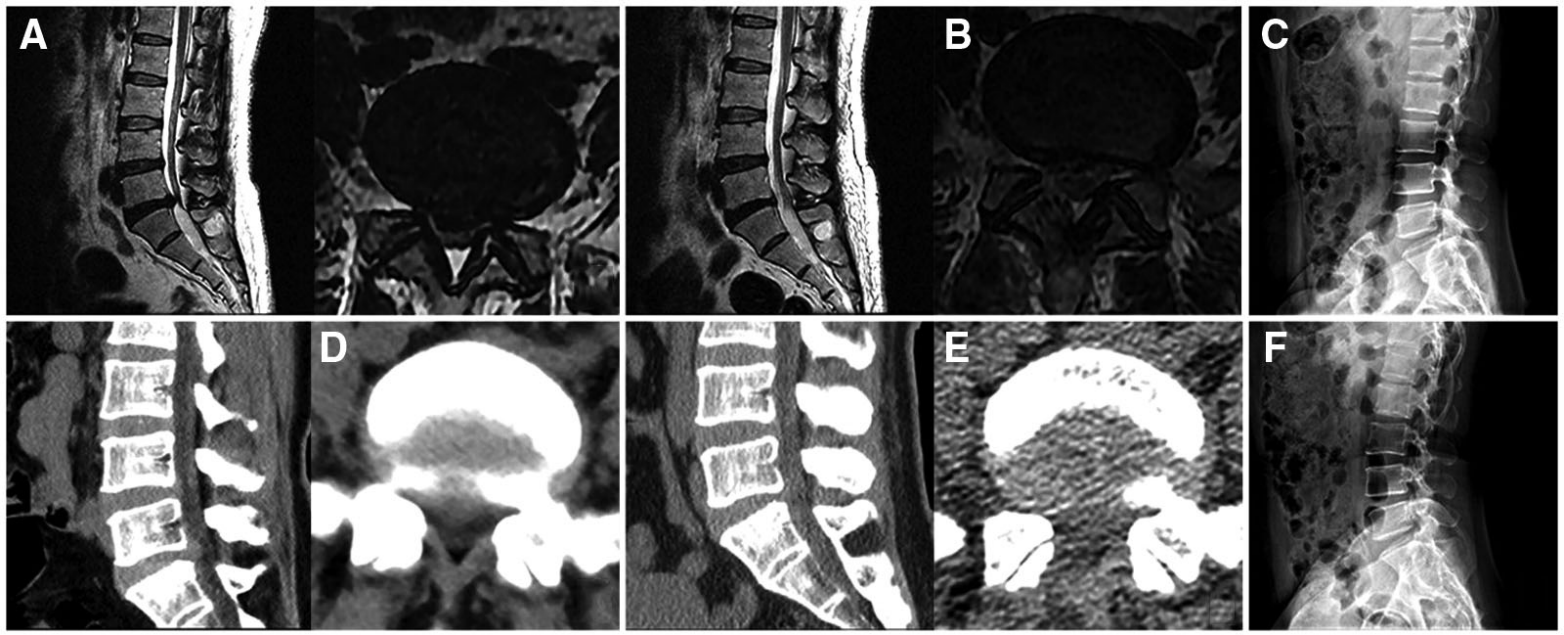
Images of a patient who underwent percutaneous endoscopic lumbar discectomy *via* the interlaminar approach. (**A**) A right L5–S1 disc herniation is shown on preoperative magnetic resonance imaging. (**B**) Postoperative imaging shows removal of the herniated disc, preservation of the annulus fibrosus, and a defect in the ligamentum flavum. (**C**) Preoperative lateral plain radiography of the lumbar spine. (**D**) L5–S1 disc herniation on preoperative computed tomography. (**E**) A window in the lamina and defects in the ligamentum flavum are seen after surgery. (**F**) Postoperative lateral plain radiography for the lumbar spine.

Patients in the PEID group underwent PEID using the following technique (5): Patients were placed in the prone position and the lumbar area disinfected and draped. The pathologic level was identified using intraoperative imaging. After administering local anesthesia, a 7 mm incision was made over the pathologic level and a dilator was inserted as close to the LF as possible. After correct placement, a grooved working cannula was inserted toward the ligament through the dilator. Then, the dilator was removed and an endoscope advanced through the cannula to visualize the fat and muscle on the surface of the LF. A nucleus pulposus forceps was used to expose the LF by clearing fat and muscle tissue away. Next, the LF was opened using a rongeur after radiofrequency coagulation to reach the spinal canal. Epidural fat was removed by the forceps and hemostasis was obtained using radiofrequency coagulation. The dural sac and nerve root were then exposed and the nerve root was separated from surrounding tissue using an adjustable nerve dissector. (ZL 2018 2 0536891.6). The grooved sheath was rotated to expose the herniated nucleus pulposus by blocking the nerve root and the dural sac, and the herniated nucleus pulposus was extracted with the forceps. Any residual loose and free nucleus pulposus was explored and removed. The annulus fibrosus and the healthy nucleus pulposus were preserved. Radiofrequency intradiscal nucleoplasty was then performed. Once lower limb symptoms and pain while coughing disappeared, the procedure was terminated. Hemostasis was then achieved and the endoscope and working cannula were removed. Figures 1, 2 illustrate a patient who underwent PEID.

**Figure 3:**
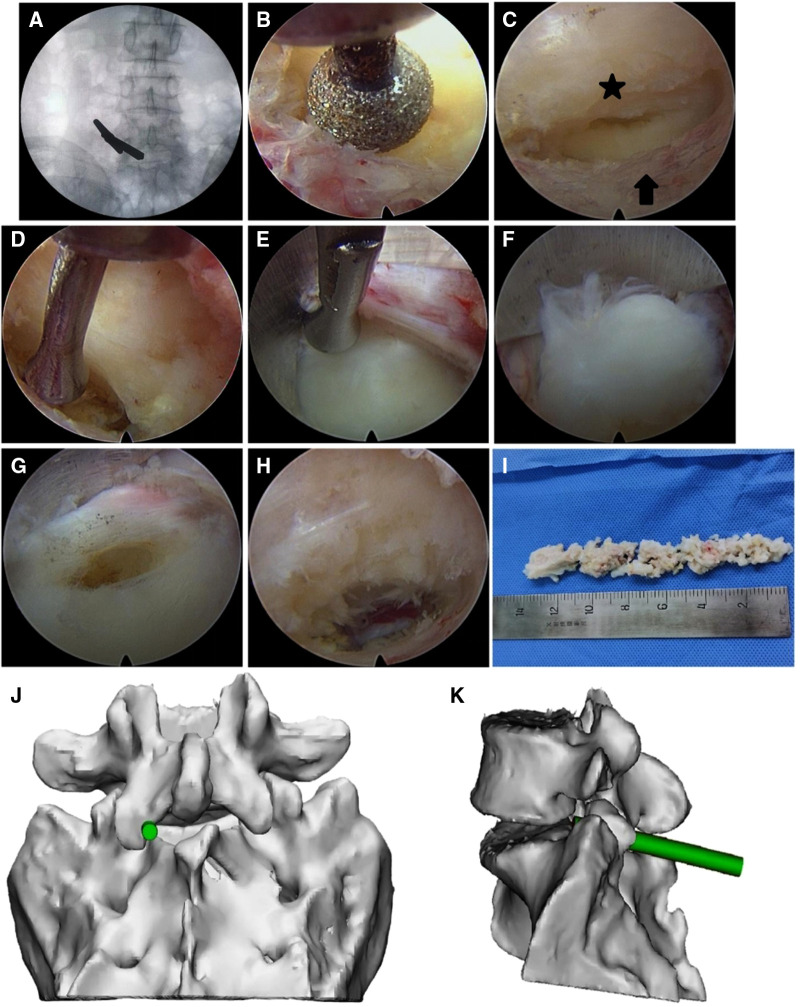
Percutaneous endoscopic lumbar discectomy *via* the medial foraminal approach in a 40-year male with L5–S1 disc herniation. (**A**) The dilator was localized using fluoroscopy before surgery and placed close to the medial articular process. (**B**) A portion of the medial articular process was removed by drilling to expose the attachment site of the ligamentum flavum. (**C**) The ligamentum flavum (star) was separated from the articular process (arrow). (**D**) The ligamentum flavum, dural sac, and nerve root were retracted medially by the adjustable nerve dissector. (**E**) The tip of the ligule groove of the working cannula was closely attached to the annulus fibrosus at the lateral disc along the medial articular process. By rotating the cannula, the ligamentum flavum, dural sac, and nerve root were moved to the center of the spinal canal. (**F**) Herniated nucleus pulposus. (**G**) The herniated nucleus pulposus was removed through the opening in the disc. (**H**) Following extraction of the herniated nucleus pulposus, the working cannula was retracted to the surface of the annulus fibrosus. The ligamentum flavum was mostly preserved, although some portions remained attached at the external margin. (**I**) The extracted nucleus pulposus tissue. (**J,K**) Schematic presentations of the working cannula in frontal (**J**) and side (**K**) views.

**Figure 4:**
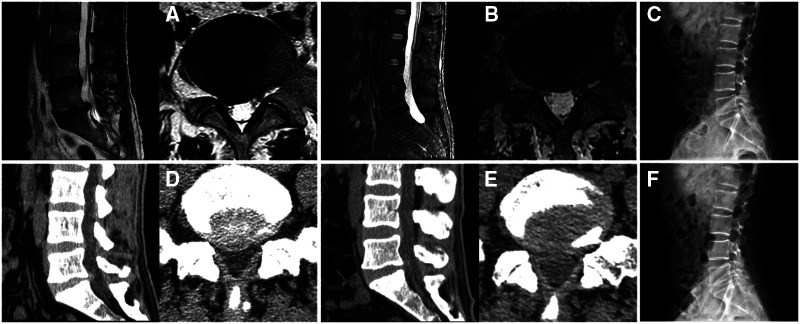
Images of a patient who underwent percutaneous endoscopic lumbar discectomy *via* the medial foraminal approach. (**A**) A right L5–S1 disc herniation is shown on preoperative magnetic resonance imaging. (**B**) Postoperative imaging shows removal of the herniated disc and preservation of the annulus fibrosus. (**C**) Preoperative lateral plain radiography of the lumbar spine. (**D**) L5–S1 disc herniation on preoperative computed tomography. (**E**) Postoperative computed tomography shows disappearance of the herniation and preservation of the ligamentum flavum. (**F**) Postoperative lateral plain radiography of the lumbar spine.

Patients in the PEMFD group underwent PEMFD using the following technique: After the same preparation described above, at the pathologic level a 7 mm incision was made over the junction between the lower margin of the upper lamina and the lower articular process. A dilator was then inserted close to the inner edge of the articular process. Once correct placement was confirmed, a grooved working cannula was inserted toward the LF through the dilator. The dilator was then removed and an endoscope advanced through the cannula. Then, the foraminal inner orifice area involving the medial lower articular process on the upper vertebral body, medial-lateral upper articular process on the lower vertebral body, and the attachment sites of the LF on the medial articular processes on the upper and lower vertebral bodies were cleaned. A drill was used to remove 1–2 mm of the osteophytic process underneath the pars interarticularis or the hypertrophic facet joint, and the attachment sites of the LF were exposed. As a result, the LF was separated from the articular process. The adjustable nerve dissector was adopted to rotate the LF together with the dural sac and nerve root medially to create a working space. The tip of the groove of the working cannula reached the surface of the annulus fibrosus at the lateral disc through the space. The cannula was then rotated and the LF, dural sac, and nerve root were gently shifted medially. During this process, the patient was questioned regarding feeling in the lower limbs. The nucleus pulposus was exposed, and the herniated portion was removed using the nucleus pulposus forceps. Residual loose and free nucleus pulposus was explored and cleaned. The annulus fibrosus and healthy nucleus pulposus were preserved. Radiofrequency intradiscal nucleoplasty was then performed. After this, the working cannula was retracted to the surface of the LF. Once lower limb symptoms and pain while coughing disappeared, the procedure was terminated. Hemostasis was then achieved and the endoscope and working cannula were removed. Figures 3, 4 illustrate a patient who underwent PEMFD.

Whether to leave the surgical drain tube depends on the amount of bleeding during operation, and this has no absolute influence on the results.

### Evaluation indices

Operation time, intraoperative bleeding volume, postoperative drainage volume, length of hospital stay, postoperative numbness, and postoperative complications were recorded. Numbness was graded using the visual analogue scale (VAS) score at baseline and 72 h after surgery. Pain VAS and Oswestry Disability Index (ODI) scores were determined at baseline and 1, 6, 12, and 24 months after surgery. At the same time, magnetic resonance imaging (MRI)、computed tomography (CT) and lumbar x-ray data before and 1, 6, 12, and 24 months after surgery were evaluated to determine LF integrity and clinical efficacy. The modified MacNab criteria were used at the 24-month follow-up to assess clinical effectiveness of surgery.

### Statistics

Statistical analyses were performed using SPSS software version 22.0 (IBM Corp., Armonk, NY, USA). Continuous and categorical variables were compared between groups using the paired t test and Fisher's exact test, respectively. *p* < 0.05 was considered significant.

## Results

Intraoperative bleeding volume (*p* = 0.004) and 24-hour postoperative drainage volume (*p* < 0.001) were significantly lower in the PEMFD group. Operation time and length of hospital stay did not significantly differ between the groups (Table 2).

**Table 2:** Operation time, intraoperative bleeding volume, postoperative drainage volume, length of hospital stay, and postoperative complications according to group.

Group	PEID group	PEMFD group	*t* value	*p* value
Case (*n*)	39	47	–	–
Operation time (min, *x* ± *s*)	77.51 ± 6.05	75.87 ± 4.52	1.438	0.154*
Intraoperative-bleeding volume (ml, *x* ± *s*)	3.56 ± 0.85	2.98 ± 0.97	2.949	0.004*
24-h drainage volume (ml, *x* ± *s*)	13.28 ± 2.68	9.98 ± 3.16	5.163	<0.001*
Hospital stay (days, *x* ± *s*)	8.29 ± 2.44	0.084	0.933*	8.26 ± 2.05
Postoperative complication				
Spinal cord injury (*n*)	0	0	–	–
Infection (*n*)	0	0	–	–
Postoperative numbness (*n*) 13	8	–	–	
Recurrent LDH (*n*)	2	2	–	–

Data are presented as means with standard deviation or numbers with percentage.

LDH, lumbar disc herniation.

*refers to comparison between PEID group and PEMFD group.

**Figure 5:**
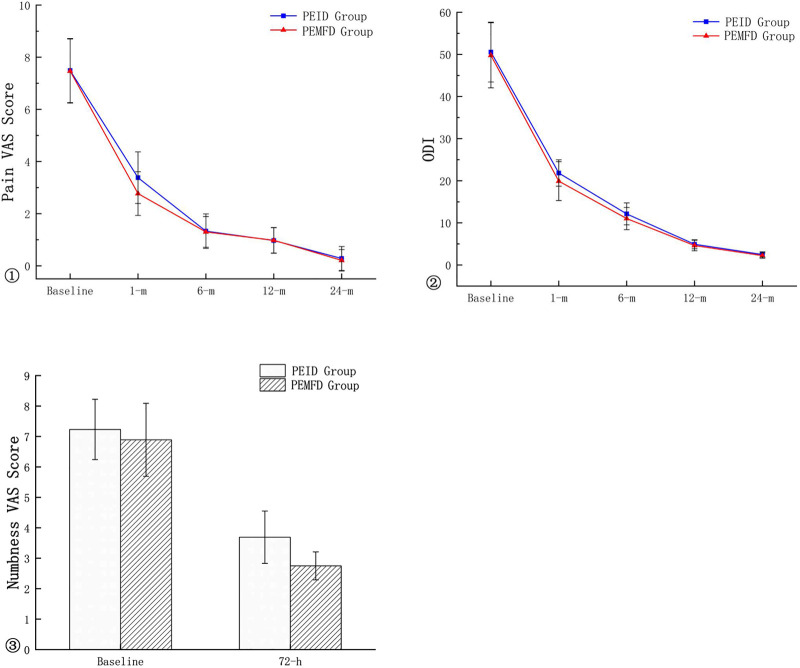
Comparison of pain, numbness VAS and oswestry disability Index scores between groups.

No patient experienced transient spinal cord injury or surgical site infection. Lower extremity numbness occurred in 13 PEID group patients and 8 PEMFD group patients; numbness gradually subsided and resolved within two to four weeks. The numbness VAS score 72 h after surgery and the pain VAS and ODI scores one month after surgery significantly differed between groups; however, pain VAS and ODI scores 6, 12, and 24 months after surgery did not (Figure 5). Recurrence occurred in two patients in each group.

At the 24-month follow-up, the proportion of patients who experienced a satisfactory modified MacNab criteria outcome (excellent, good, or fair) did not significantly differ between the groups (*χ*^2^ = 1.833, *p* = 0.608; Table 3).

**Table 3:** Modified MacNab outcome according to group.

Group	Excellent	Moderate	Good	Satisfactory rate (%)	*χ*^2^ value	*p* value
PEID group (*n*)	29	6	4	89.74	1.833	0.608
MF-PELDgroup (*n*)	39	5	3	93.62		
Total	68	11	7	–	–	–

## Discussion

The minimally invasive PEID allows removal of herniated nucleus pulposus after separating the LF, dural sac, nerve root, and soft tissue and is performed using endoscopy with patients in the prone position. It is superior to the conventional open approach, as it is associated with less trauma, less intraoperative blood loss, faster recovery, and fewer postoperative complications (15). Furthermore, satisfactory efficacy of this approach has been demonstrated in a clinical setting. However, this approach requires partial laminectomy and removal of a portion of LF to create a working space. Because the LF is opened, bleeding may occur from the abundant venous plexus on the surface of the dural sac and postoperative adhesions may develop. Epidural fibrosis and scar formation may also occur (16). Preservation of the LF can decrease postoperative adhesions of the dural sac and nerve root, as the LF can block scar growth by serving as a barrier (17). The LF is located between two adjacent laminae and comprises a portion of the posterior spinal canal wall. It can be divided into overlapping superficial and deep layers. In the superficial layer, the upper edge is attached to the anterior middle-lower edge of the upper lamina, the external side is attached to the root of the lower articular process, and the inferior edge is attached to the upper and posterior side of the lower lamina. In the deep layer, the upper edge is attached to the anterior upper lamina and the inferior edge to the upper lower lamina (18). The LF is composed of elastic fibrous tissue and serves as a structure responsible for limiting excessive spinal flexion and maintaining the standing posture (18, 22). Therefore, preserving the LF during the surgery can maintain spinal stability, control interspinal movement, and keep the surface of the posterior dural sac smooth (19). The LF also serves as a natural barrier that separates the dura, nerve root, and subdural fat from surrounding tissues (20). Considering this, the LF should be preserved during surgery if possible in an attempt to reduce iatrogenic dural sac injuries and prevent postoperative adhesions.

**Figure 6:**
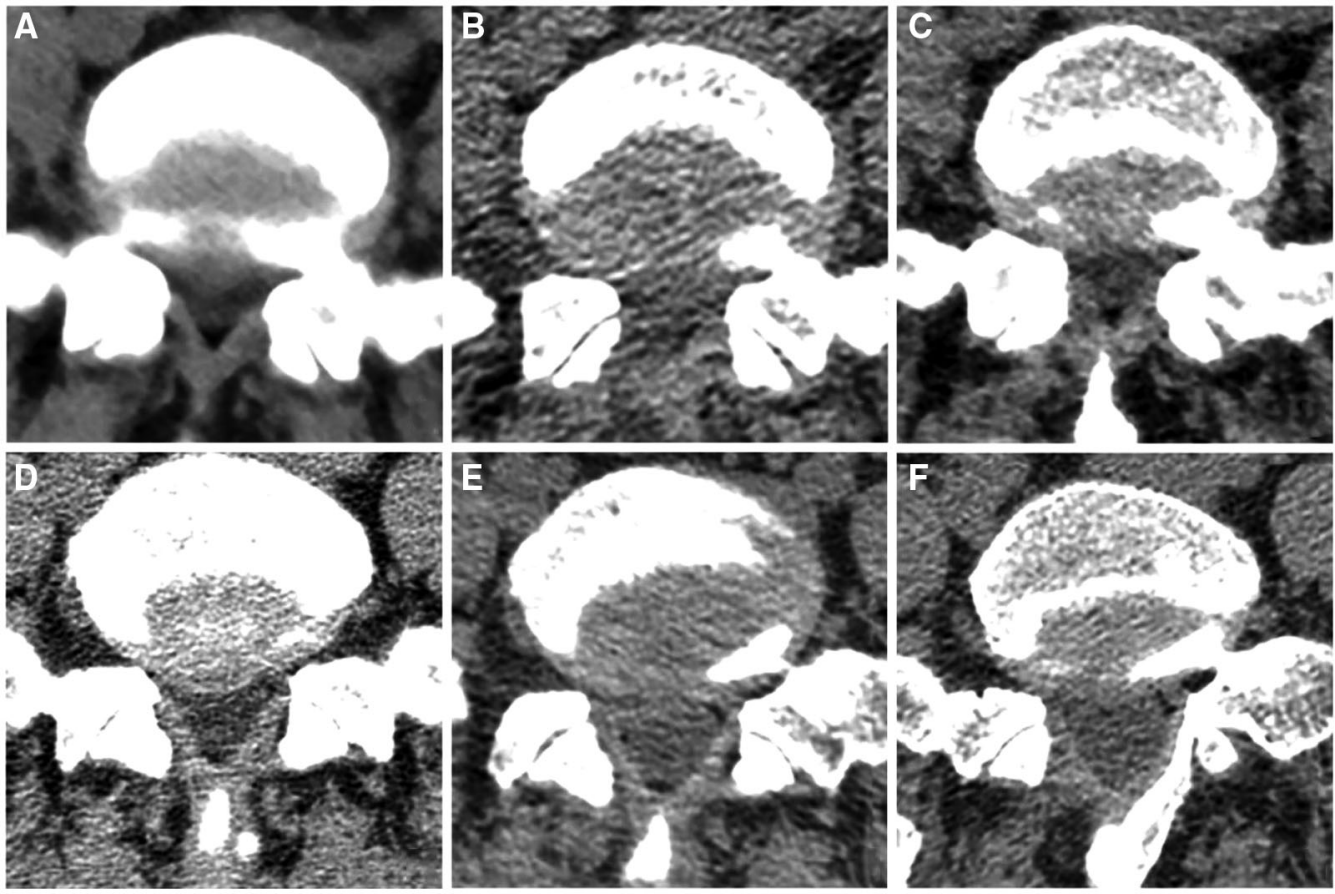
Integrity of the ligamentum flavum on computed tomography before and after surgery in PEID group (**A–C**) and MF-PELD group (**D–F**). (**A**) Preoperative ligamentum flavum. (**B**) Damaged ligamentum flavum after surgery. (**C**) Scar repair in the ligamentum flavum defect six months later. (**D**) Preoperative ligamentum flavum. (**E**) Ligamentum flavum after surgery has been separated from its attachment site. (**F**) Normal-appearing ligamentum flavum with clear layers six months after surgery.

The PEMFD technique preserves the LF to a considerable extent. Figure 6 shows the integrity of the LF on computed tomography before and after surgery in the PEID and PEMFD groups. Lee et al. (21) divided the foraminal region into three parts (medial, central, and lateral) with boundaries defined by the medial and external links between the upper and lower pedicles. The foramen opens medially into the spinal canal and is bounded anteriorly by the posterior medial intervertebral disc, posteriorly by the medial upper articular process, superiorly by the inferior-medial upper pedicle, and inferiorly by the upper-medial part of the lower pedicle. The LF can be successfully separated from the articular process without causing any damage to it; therefore, it can be shifted medially together with the dural sac. This technique requires fewer operative steps and preserves the integrity of the LF and dural sac.

The PEMFD technique has the following advantages: First, the MF technique only removes the osteophytic process underneath the pars interarticularis or the hypertrophic facet joint, which separates the LF and articular process from the external side while preserving the LF. This can prevent posterior epidural scar tissue from invading the spinal canal. Furthermore, partial removal of the LF, which can lead to formation of scar and adhesions surrounding the dura mater, is avoided (22). Second, the LF remains well preserved, which assists in maintaining mechanical stability. Third, the LF protects the nerve root and dural sac when they are all moved together during the operation: the dural sac is less likely to be damaged and the risk of cerebrospinal fluid leakage is reduced. All lumbar discectomies are associated with risk of LDH recurrence that may require a second operation. Repeat surgery is technically difficult and associated with greater complication risk because of the presence of scarring and adhesions in the spinal canal. With the PEMFD technique, the LF is preserved, which reduces postoperative scar and adhesion formation. Reoperation after PEMFD should have a lower incidence of dural sac injury. In this study, patients who underwent PEMFD had easier operations and less bleeding than those who underwent PEID. However, length of hospital stay and incidence of postoperative complication did not significantly differ. At the 24-month follow-up, VAS and ODI scores and the proportion of patients who experienced a satisfactory outcome were similar between the two groups, indicating similar medium-term outcomes.

Lower extremity numbness occurred in 13 PEID group patients and 8 PEMFD group patients. We believe the incidence of numbness is related to two factors: 1) nerve root edema and compression by hematoma; and 2) greater LF damage in PEID group patients. Patients in the PEID group experienced greater intraoperative dural sac stimulation, which caused a higher incidence of postoperative pain and numbness; however, this symptom gradually resolved with pharmacologic treatment.

**Figure 7:**
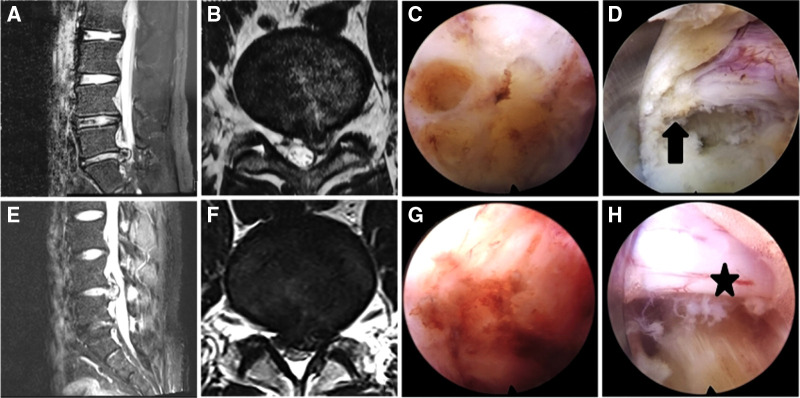
Magnetic resonance imaging (MRI) and intraoperative photographs in a PEID group patient with recurrent disc herniation (**A–D**) and a MF-PELD group patient with recurrent disc herniation (**E–H**). (**A,B**) Left L4–5 disc herniation is shown on MRI. (**C**) Scarring is present and the local anatomy is poorly defined. (**D**) The surface of the nerve root appears rough and is surrounded by scar (arrow). (**E,F**) Left L4–5 disc herniation is shown on MRI. (**G**) Less scarring is seen during surgery. (**H**) The nerve root has clear boundaries and is surrounded by little scar (star) and no adhesive bands.

Two patients in each group experienced LDH recurrence. All performed heavy manual work within 3 months of surgery, which was the probable cause. In the repeat operations, severe scarring and adhesions were observed in the PEID group patients, which increased the technical difficulty. The PEMFD group patients had less scarring and anatomic structures were well-defined Figure 7 shows magnetic resonance imaging and intraoperative photographs in two patients who underwent surgery for recurrent disc herniation. Therapeutic outcomes in both groups were acceptable, which infers that PELD is an effective treatment for LDH, even recurrent ones.

Despite the advantages of the MF technique, several disadvantages should be mentioned. This approach should be performed with caution in patients with LF hypertrophy and lumbar spinal stenosis. Translocation of a hypertrophic LF along with the dural sac and nerve could crush the nerve and dural sac within the spinal canal and cause injury. In this circumstance, the spinal canal should be expanded first be removing bone or the LF should be partially excised to allow for smooth movement of the dural sac and nerve root. Operators must understand the anatomic structure of the foraminal inner opening and have competent endoscopic skills. However, because the operation is fairly simple and straightforward, the learning curve is relatively short.

## Conclusion

PEID and PEMFD have similar short- and medium-term results and are both safe and effective for treating LDH. However, PEMFD has several advantages: simplicity, lower bleeding volume, and preservation of the LF. LF preservation reduces the probability of iatrogenic dural sac injury and postoperative epidural adhesions.

## Data Availability

The raw data supporting the conclusions of this article will be made available by the authors, without undue reservation.
